# Engineering Hierarchical Cellulose Aerogel Networks Toward Decoupled Heat Transfer and Enhanced Multi-Phase Fire Safety

**DOI:** 10.3390/ma19143106

**Published:** 2026-07-20

**Authors:** Lei Chen, Haiyan Wang, Wei Ding, Xiaodong Qian, Congling Shi, Ye-Tang Pan, Mei Wan, Jingyun Jing, Yanan Hou

**Affiliations:** 1 School of Emergency Management and Safety Engineering, China University of Mining Technology (Beijing), Xuyuan Road Ding 11, Haidian District, Beijing 100012, China; cl1902467747@126.com (L.C.); whyhyp@163.com (H.W.); shicl@chinasafety.ac.cn (C.S.); 2 China Academy of Safety Science and Technology, Shuangying Road, Beijing 100012, China; wan1112mei@163.com (M.W.); bdqyjjy@163.com (J.J.); hhouyn@chd.edu.cn (Y.H.); 3 National Engineering Research Center of Flame Retardant Materials, School of Materials Science & Engineering, Beijing Institute of Technology, Beijing 100081, China; pyt@bit.edu.cn; 4 School of Geological Engineering and Geomatics, Chang’an University, Xi’an 710018, China

**Keywords:** cellulose aerogels, hierarchical network, heat transfer regulation, flame retardancy, thermal insulation, fire safety

## Abstract

**Highlights:**

- Bioinspired cellulose aerogels with a web-like network were prepared.
- Dual fillers acted as structural regulators and flame-retardant components.
- The composite aerogel achieved a low thermal conductivity of 35 mW·m^−1^·K^−1^.
- Synergistic multi-phase flame retardance suppressed heat and mass transfer.

**Abstract:**

Cellulose-based aerogels are promising sustainable thermal-insulation materials, but their practical application is often limited by insufficient mechanical robustness and intrinsic flammability. Herein, a multiscale network-engineering strategy is proposed to fabricate a cellulose-based composite aerogel integrating structural stability, thermal insulation, and fire safety. By synergistically introducing in situ generated aluminum trihydroxide (ATH) and microencapsulated APP@ATH–MEL into the cellulose scaffold, the flame-retardant components function not only as active fire-safety agents but also as structural regulators that promote the formation of a highly interconnected hierarchical framework. This regulated architecture enhances interfacial interactions, improves load-transfer efficiency, suppresses structural collapse during freeze-drying, and introduces tortuous pathways and abundant interfaces for heat-transfer regulation. As a result, the optimized composite aerogel exhibits a low thermal conductivity of 35 mW·m^−1^·K^−1^ together with improved compression resistance. Thermal analysis reveals a reduced mass-loss rate and increased char yield, while cone calorimetry confirms suppressed heat release, reduced gaseous emissions, and improved residue stability. The enhanced fire safety is attributed to a synergistic multi-phase mechanism involving endothermic shielding, gas-phase dilution, condensed-phase char formation, and inorganic-residue reinforcement, which collectively inhibit heat and mass transfer during combustion. This work provides an effective strategy for the design of lightweight, mechanically robust, and fire-safe cellulose-based composite aerogels for advanced thermal-insulation applications.

## 1. Introduction

Lightweight thermal-insulation materials with reliable fire safety are increasingly demanded in engineering sectors such as building envelopes, transportation systems, energy facilities, and aerospace structures. In these scenarios, insulation layers may encounter elevated temperatures or accidental fire exposure [1,2]. Under such conditions, insufficient flame retardancy or structural instability can lead to rapid degradation and serious safety risks [3,4,5]. Developing materials that combine low density [6], low thermal conductivity [2,7], mechanical stability [8], and effective fire resistance [9,10] therefore remains a persistent challenge. Cellulose-based aerogels, in particular, offer ultralow density and high porosity, together with interconnected three-dimensional frameworks. These features are beneficial for limiting heat transfer while maintaining lightweight characteristics. Yet, despite these advantages, several limitations remain. The organic nature of cellulose and its highly porous structure render these aerogels inherently flammable and mechanically fragile [11,12]. They are also prone to shrinkage or collapse under thermal stress. Open pore channels further facilitate oxygen diffusion and volatile transport during combustion, which can accelerate flame propagation and weaken the protective effect of the residual layer.

Various flame-retardant strategies have been explored to address these issues. These include the incorporation of inorganic fillers, phosphorus-containing additives, and hybrid systems. Metal hydroxides, for example, can absorb heat and release nonflammable species upon decomposition. Phosphorus- and nitrogen-containing compounds, on the other hand, promote char formation and contribute to gas-phase dilution. However, conventional additive-based approaches often require [13,14] relatively high loadings to achieve sufficient fire protection [15,16]. This can increase density and compromise thermal-insulation performance. It may also affect structural integrity. In addition, limited interfacial compatibility and uneven particle distribution can lead to aggregation, pore collapse, and weakened mechanical properties during gelation and freeze-drying [17,18]. Recent studies suggest that performance improvements depend not only on the choice of flame-retardant components but also on how the porous framework is constructed. Network regulation and hierarchical structural design play a key role [12,19]. A well-organized three-dimensional framework can stabilize pore structures, improve load transfer, and reduce thermal shrinkage. It can also support the formation of more continuous protective residues during combustion. In cellulose-based aerogels, this aspect is particularly important, as fire performance is closely linked to the integrity of the porous network under thermal exposure. Nevertheless, achieving a stable and multifunctional flame-retardant structure remains challenging. Many reported approaches involve complex processing routes or insufficient control over particle distribution [13,20], and the relationship between pore architecture, heat transfer, and combustion behavior is not always clearly understood [2,21,22,23]. Among available flame-retardant components, aluminum trihydroxide (ATH) is attractive due to its low cost [24,25,26], low toxicity, and endothermic decomposition behavior, which releases water vapor under heating. However, its effectiveness in lightweight porous systems is often limited unless used at relatively high contents [10,27,28]. In contrast, phosphorus- and nitrogen-containing compounds such as ammonium polyphosphate (APP) and melamine (MEL) can enhance condensed-phase carbonization and release nonflammable gases [29,30]. Combining ATH with phosphorus–nitrogen chemistry therefore offers a feasible route to synergistic fire protection, provided that these components can be uniformly incorporated into the cellulose scaffold and contribute to structural regulation [31,32].

Herein, we report a cellulose-based composite aerogel fabricated through a synergistic pre-sol–gelation and sol-aging strategy, in which in situ precipitated aluminum trihydroxide (ATH) and microencapsulated APP@ATH–MEL are integrated into the cellulose scaffold. Unlike conventional additive-based approaches, these functional components serve not only as flame-retardant agents but also as structural regulators that help generate abundant interaction sites throughout the framework. The resulting particulate-reinforced hierarchical network improves structural stability during freeze-drying, enhances resistance to thermally induced collapse, and promotes more efficient regulation of heat and mass transfer. Consequently, the composite aerogel exhibits improved surface characteristics, enhanced compression resistance, delayed thermal degradation, reduced mass-loss rate, and increased char yield. TG–FTIR and cone-calorimetry results further indicate that the improved fire performance originates from a coupled condensed-phase and gas-phase mechanism involving endothermic shielding, volatile dilution, and residue stabilization. More importantly, this work highlights how network-topology regulation can be combined with multi-phase flame-retardant action to develop lightweight, fire-safe cellulose-based composite aerogels for demanding thermal-insulation applications.

## 2. Experiment and Material

### 2.1. Material

Carboxymethyl cellulose (CMC, viscosity 600–3000 mPa·s) was purchased from Sinopharm Chemical Reagent Co., Ltd. (Shanghai, China). Aluminum chloride hexahydrate (AlCl_3_·6H_2_O), ammonium hydroxide, melamine (MEL), and ammonium polyphosphate (APP, 10 < n < 90) were also obtained from Sinopharm Chemical Reagent Co., Ltd. (Shanghai, China). Tetraethoxysilane (TEOS), the silane coupling/crosslinking agent KH-560 (Propyltrimethoxysilane), ethanol, polyethylene glycol, and ammonia solution (28–33 wt%) were purchased from Macrolin Biotechnology Co., Ltd. (Shanghai, China). Distilled water was supplied by Foshan Tianqing Water Treatment Co., Ltd. (Foshan, China). All chemicals were used as received without further purification unless otherwise specified.

### 2.2. Preparation of Cellulose-Based Aerogel and ATH/CMC Precursor

CA and CA/ATH composite aerogels were prepared from sodium carboxymethyl cellulose (CMC). Briefly, 0.8 g of CMC was dissolved in 40 mL of water at 25 °C under continuous stirring until a homogeneous solution was obtained. For CA, the CMC solution was directly poured into customized molds, frozen overnight, and freeze-dried for 72 h. For CA/ATH, a mixed solution containing 10 wt% AlCl_3_·6H_2_O and 2 mL ammonia water was uniformly added to the homogeneous CMC solution under continuous stirring at 25 °C. After complete mixing, the precursor solution was poured into customized molds, frozen overnight, and freeze-dried for 72 h to obtain the CA/ATH composite aerogels.

### 2.3. Preparation of Flame-Retardant Suspension

To prepare the flame retardant, this study adopted a similar method and referred to previous works [33,34,35]. The flame-retardant precursor suspension was prepared according to a modified procedure based on previous reports. Briefly, 5 g of APP and 0.34 g of CTAB were dispersed in an ethanol/water mixed solvent (water/ethanol = 5:3 by volume) containing 2 mL ammonia solution to form a uniform suspension. Under vigorous stirring, 1 mL TEOS, 4 g AlCl_3_·6H_2_O, and KH-560 (or the corresponding silane reagent, as applicable) were added sequentially. The mixture was then maintained in a 60 °C water bath for 24 h. After reaction, the product was collected by centrifugation at 10,000 rpm for 10 min, washed twice with distilled water and absolute ethanol, and dried at 80 °C for 24 h to obtain APP@ATH. Subsequently, 2 g of MEL was dissolved in 100 mL ultrapure water, followed by the addition of 2 mL phytic acid (PA) under vigorous stirring. Finally, 1 g of APP@ATH was added to obtain the APP@ATH–MEL suspension.

### 2.4. Preparation of Flame Retardant Aerogels

To prepare the cellulose-based composite aerogels, 20 mL of cellulose sol (2 wt%) was transferred into a mold, followed by the addition of 4 mL of the corresponding flame-retardant suspension. The mixture was heated in a 60 °C water bath and stirred at 1000 rpm for 30 min to promote homogeneous dispersion and precursor interaction. The obtained wet gel or gel-like precursor was then subjected to freezing and freeze-drying to yield the final cellulose-based composite aerogel. According to the different formulations, samples such as CA/MEL and CA/APP@ATH–MEL were prepared for comparison.

### 2.5. Characterizations

***Scanning Electron Microscopy (SEM)***

The morphology and microstructure of the aerogels were characterized by scanning electron microscopy (SEM, Phenom XL G2, Thermo Fisher Scientific, Eindhoven, The Netherlands). Prior to imaging, all specimens were sputter-coated with gold using an SBC-12 sputter coater (Beijing KYKY Technology Co., Ltd., Beijing, China) at 10 mA for 60 s. SEM observations were conducted at an accelerating voltage of 5 kV. Elemental distribution was analyzed by energy-dispersive spectroscopy (EDS) attached to the SEM instrument at an accelerating voltage of 20 kV.

***Energy-Dispersive Spectroscopy (EDS)***

Elemental composition analysis was performed in conjunction with SEM (Phenom XL G2) using an energy-dispersive spectrometer (Thermo Scientific, Eindhoven, The Netherlands) under an accelerating voltage of 20 kV. The scan rate was maintained at 20,692 counts per second to ensure optimal spectral acquisition for compositional mapping.

***Thermal Stability***

Thermal stability was evaluated using a TGA 550 thermogravimetric analyzer (TA Instruments, New Castle, DE, USA). Approximately 3–6 mg of sample was heated from 50 to 800 °C at 20 °C·min^−1^ under nitrogen atmosphere. TG–FTIR analysis was conducted using a coupled PerkinElmer TGA 4000 and SP2 FTIR system at a heating rate of 10 °C·min^−1^ over the range of 50~800 °C (PerkinElmer, Shelton, CT, USA).

***Thermogravimetric Analysis Coupled with Fourier Transform Infrared Spectroscopy (TG-FTIR)***

TG-FTIR analysis was conducted to qualitatively and quantitatively characterize volatile and decomposition products released during heating. The coupled system consisted of a PerkinElmer TGA 4000 with an SP2 FTIR unit. The heating program was set to 10 °C·min^−1^ from 50 °C to 800 °C. FTIR spectra were collected over the range 400–4000 cm^−1^ at a resolution of 2 cm^−1^.

***Flame-Retardant Performance Analysis***

All samples were kept in a drying oven for 24 h to eliminate internal moisture and residual water content. Cone calorimetry tests were performed on a Fire Testing Technology (FTT) apparatus (Fire Testing Technology Ltd., East Grinstead, UK) under an external heat flux of 35 kW·m^−2^. Test specimens measured 100 mm × 100 mm × 20 mm.

***Surface Area Analysis***

Specific surface area and pore structure measurements were conducted using a Micromeritics ASAP 2020 instrument (Micromeritics, Norcross, GA, USA). Nitrogen adsorption–desorption experiments were carried out at 200 °C after degassing for 8 h under vacuum.

***Statistical Analysis and Imaging***

All measurements were performed in triplicate, and results are reported as mean ± standard deviation. Statistical data analysis was conducted using Origin software (2025 sp). Optical images of aerogels, cone calorimeter residues, and cellulose combustion processes were captured using a Canon EOS RP camera (Canon Inc., Tokyo, Japan). Burning process videos and photographs were cropped and edited using Adobe Premiere Pro 2025, without any additional image enhancement or manipulation.

## 3. Results and Discussion

The enhancement of aerogel performance through crosslinking is attributed to the formation of stable covalent/ionic bonds within the three-dimensional polymer network. These bonds immobilize the polymer chains, restrict segmental motion, and prevent structural collapse under mechanical, thermal, or solvent stress. Furthermore, incorporation of functional crosslinkers containing phosphorus, nitrogen, or silicon introduces an inherent flame-retardant mechanism via char promotion and barrier layer formation, while hydrophobic modification through silane crosslinkers suppresses moisture uptake. Such structural stabilization mechanisms synergistically improve mechanical integrity, thermal stability, specific surface area, and functional reliability of bio-based aerogels under diverse operating conditions.

### 3.1. Analysis of Micromorphology and Performance

#### 3.1.1. Morphology and Microstructure

**Figure 1:**
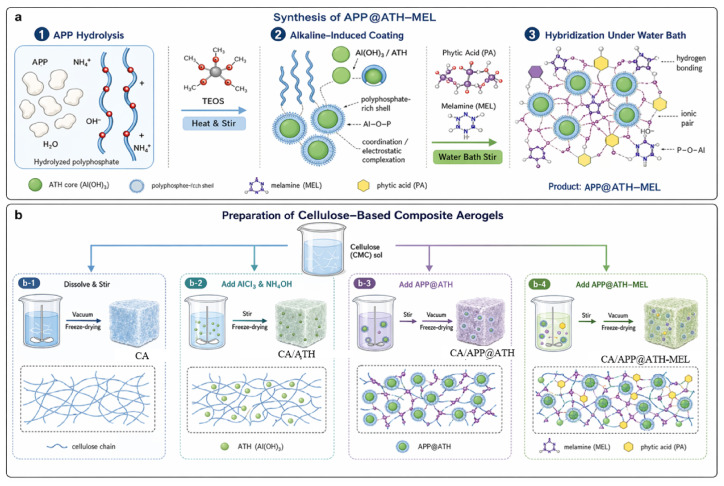
Schematic illustration of the preparation route for the flame-retardant components and cellulose-based composite aerogels: (**a**) synthesis of the APP@ATH-related precursor system; (**b**) preparation of the cellulose-based aerogel and composite aerogels through precursor mixing, gelation, freezing, and freeze-drying; (**b-1**–**b-4**) are schematic illustrations displaying the fabrication process and internal crosslinking structure of CA, CA/ATH, CA/APP@ATH, and CA/APP@ATH-MEL.

The synthesis of the flame-retardant aerogels involves multiple chemical interactions that strongly regulate the rheological behavior of the precursor sols (Figure 1b). The sol was fixed during freezing, and the internal ice crystals were subsequently removed during drying while the porous structure was retained, resulting in the formation of the aerogel. As evidenced by SEM observations and BET analysis, the introduction of the dual-filler system progressively enhances both physical and chemical crosslinking, driving the structural evolution from relatively ordered lamellar morphologies in pristine CA to a highly interconnected network with improved pore homogeneity. Correspondingly, the specific surface area increases markedly from 3.30 m^2^·g^−1^ for pristine CA to 24.76 m^2^·g^−1^ for the CA/APP@ATH–MEL composite aerogel. The increase in specific surface area is mainly associated with the formation and preservation of micro-/mesoporous structures during freeze-drying, rather than being solely attributed to network crosslinking. The incorporation of APP@ATH–MEL may also alter the original macroporous framework of pristine CMC, leading to a more hierarchical pore architecture. It should be noted that the thermal conductivity of aerogels is governed not only by specific surface area, but also by apparent density, solid fraction, pore-size distribution, and the continuity of the solid framework. Therefore, the low thermal conductivity of the composite aerogel should be understood as the combined result of hierarchical pore regulation, tortuous heat-transfer pathways, restricted gas convection, interfacial scattering, and framework preservation. Compared with pristine CA, the incorporation of APP@ATH–MEL introduces abundant adsorption, coordination [36,37], and hydrogen-bonding sites into the cellulose scaffold framework (CSF), which facilitates dense network formation during ultrasonic dispersion and subsequent sol–gel aging. During aging, condensation reactions between APP@ATH–MEL and the functional groups of the CSF further reinforce network connectivity, resulting in a mechanically strengthened framework with improved load-transfer capability. The formation of milky precursor sols after APP@ATH–MEL addition further supports the presence of enhanced colloidal interactions and improved dispersion stability. Meanwhile, the infiltration and immobilization of flame-retardant components within the CSF ensure their uniform structural integration, which is essential for constructing a continuous protective network and enabling the rapid activation of multi-phase flame-retardant mechanisms during combustion [21,38].

**Figure 2:**
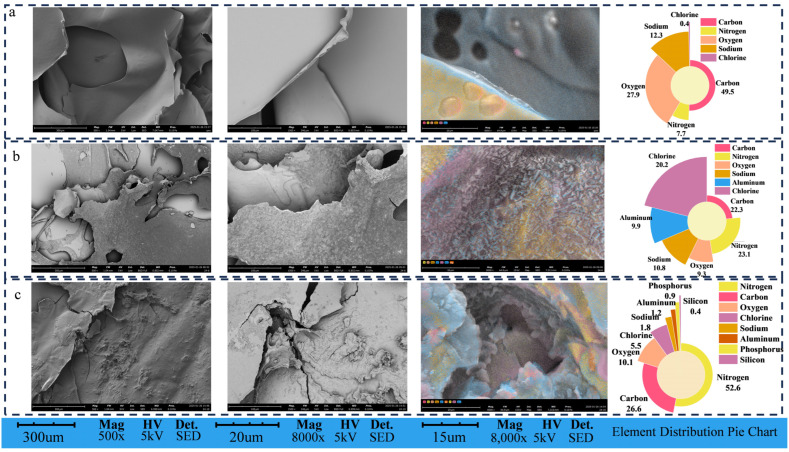
SEM images of (**a**) pristine CA, (**b**) CA/ATH, and (**c**) CA/APP@ATH–MEL aerogels at different magnifications, showing the evolution of the lamellar framework after the introduction of ATH and APP@ATH–MEL. Corresponding elemental mapping images confirm the distribution of Al, P, and N within the composite aerogel scaffold. Au was excluded from the elemental composition because the samples were sputter-coated with gold before SEM-EDS analysis for conductivity.

The microstructural evolution of the aerogels was further examined by SEM and elemental mapping (Figure 2). At low magnification (500×, Figure 2a–c), pristine CA display relatively smooth and ordered lamellar morphologies generated during directional freeze-drying. After the incorporation of ATH and APP@ATH–MEL, the microstructure becomes increasingly intricate and heterogeneous, accompanied by enhanced surface roughness and structural complexity. At intermediate magnification (1000×), the composite aerogels exhibit pronounced surface folding, granular features, and markedly improved interlayer connections, indicating strengthened interactions between adjacent lamellae. At higher magnification (8000×), fine particulate bridges spanning neighboring lamellae are clearly observed in the CA/APP@ATH–MEL aerogel, whereas such features are much less developed in pristine CA and CA/ATH. These bridging structures highlight the critical role of APP@ATH–MEL in reinforcing interlamellar connectivity and constructing a three-dimensional interconnected framework. Elemental mapping at the same magnification confirms the homogeneous distribution of Al, P, and N within the interlamellar regions, demonstrating that ATH and APP@ATH–MEL are not merely dispersed additives but active participants in network formation. The formation of such continuous crosslinking domains is crucial for maintaining structural integrity and functional performance. Mechanistically, this structural evolution can be attributed to enhanced multiscale crosslinking induced by the dual-filler system. In situ generated ATH acts as rigid inorganic nodes that promote short-range coordination interactions with cellulose chains, while APP@ATH–MEL supplies abundant functional groups capable of hydrogen bonding, ionic interactions, and condensation reactions. Together, these effects strengthen intermolecular associations within the CSF, improve resistance to ice-crystal-induced damage during freeze-drying, suppress pore collapse, and enable the formation of stable bridging structures between adjacent lamellae. In contrast, pristine CA relies mainly on weak hydrogen bonding and van der Waals interactions, which are insufficient to resist ice-crystal growth and therefore lead to irregular pore morphologies and structural defects. These SEM observations suggest a pronounced evolution in microstructure after the incorporation of ATH and APP@ATH–MEL, manifested by rougher lamellar surfaces, increased particulate decoration, and improved interlamellar contact. However, these features should be interpreted as morphological evidence of network regulation rather than direct proof of chemical crosslinking. The structural changes may also be associated with altered sol viscosity, particle–matrix interactions, and modified gelation behavior during freeze-drying.

#### 3.1.2. Analysis of Micromorphology and Pore Structure

**Figure 3:**
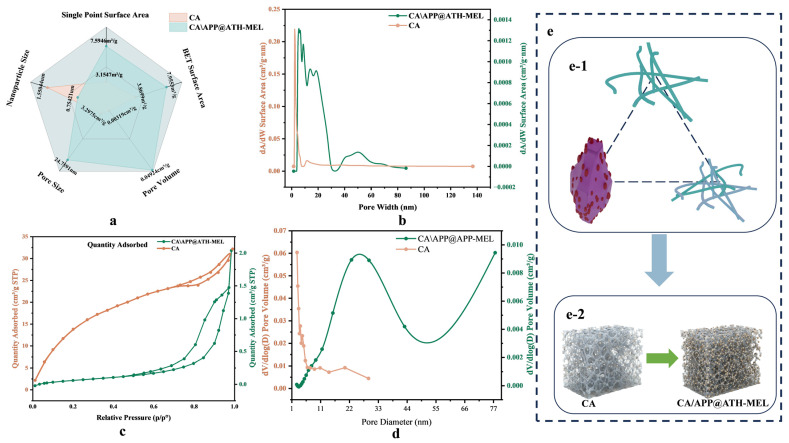
(**a**–**c**) Representative microstructural images of pristine CA, CA/ATH, and CA/APP@ATH–MEL aerogels, illustrating differences in pore organization and framework morphology. (**d**) Nitrogen adsorption–desorption isotherms and (**e**) corresponding pore-size distribution curves, showing the evolution of surface area and pore characteristics after the introduction of ATH and APP@ATH–MEL.

SEM characterization (Figure 3a–c) reveals that pristine CA exhibit relatively well-aligned lamellar structures; however, numerous irregular pores are present between adjacent layers because of insufficient crosslinking. Although the cellulose scaffold framework (CSF) is inherently stable, the weak hydrogen bonding and van der Waals interactions among cellulose chains are insufficient to withstand the disruptive stresses induced by ice-crystal growth during freeze-drying, resulting in local chain rupture, pore collapse, and poorly supported irregular cavities. In contrast, the introduction of metal-ion-mediated crosslinking promotes the in situ formation of ATH species under alkaline conditions, generating short-range crosslinked structures that strengthen both inter- and intramolecular interactions among cellulose chains. This reinforced framework effectively resists the stresses associated with ice crystallization during freezing and drying. In the CA/APP@ATH–MEL system, melamine molecules further participate in intermolecular interactions with cellulose chains, thereby intensifying interchain connectivity. At the same time, uniformly dispersed nanoparticles act as structural fillers, suppressing lamellar collapse caused by water loss and ice-crystal sublimation and preserving stable pore-bridging structures between adjacent cavities. Morphologically, pristine CA surfaces appear relatively smooth and contain large voids, whereas CA/ATH exhibits a more intricate crosslinked architecture decorated with embedded particulates. After the incorporation of APP@ATH–MEL, the aerogel surface becomes densely populated with fine particulate features and additional vertical supports, reflecting the structural integration of the flame-retardant components. This evolution favors the formation of a robust three-dimensional porous framework at comparable bulk density, which strongly influences the adsorption–desorption behavior.

Nitrogen adsorption–desorption analysis further substantiates these structural differences (Figure 3d,e). Pristine CA exhibit a BET surface area of 3.86 m^2^·g^−1^, an average pore size of 3.29 nm, and a pore volume of 0.0032 cm^3^·g^−1^. After the introduction of ATH and APP@ATH–MEL, the BET surface area initially decreases to 1.44 m^2^·g^−1^, while the average pore size increases to 5.3595 nm and the pore volume decreases to 0.0019 cm^3^·g^−1^. This initial decrease in accessible surface area can be attributed to partial pore blocking and local structural densification caused by the anchoring of inorganic and hybrid particles within the CSF. The pore size obtained from nitrogen adsorption–desorption represents adsorption-accessible mesopores/nanopores, while the SEM images mainly show micrometer-scale lamellar cavities and macroporous channels. Thus, the two results characterize different levels of the hierarchical pore structure rather than the same pore population.

After optimization of the sol–gel process, including precursor pre-dispersion, controlled sol aging, and scaffold stabilization, the surface characteristics of the composite aerogel are significantly improved. Under these optimized conditions, the specific surface area of CA/APP@ATH–MEL increases from 3.86 m^2^·g^−1^ for pristine CA to 7.95 m^2^·g^−1^, corresponding to an approximately 200% increase in single-point surface area (from 3.15 to 7.59 m^2^·g^−1^). This result indicates that the dual-filler strategy involves a competitive process between pore blocking and network stabilization: although filler incorporation initially reduces accessible pore volume, the establishment of a robust multiscale crosslinked framework effectively suppresses structural collapse during freeze-drying and ultimately generates a more stable hierarchical pore architecture.

Mechanistically, the sol–gel pathway employed in this work (Figure 1a) relies on the formation of weakly basic ATH species in the alkaline cellulose environment, which coordinate with cellulose chains to construct a rigid scaffold. Simultaneous metal–ligand interactions and hydrogen bonding anchor ATH particles within the CSF, while ultrasonic treatment and high-temperature aging improve precursor dispersion, remove excess NH_3_·H_2_O, and stabilize the local chemical environment [2,5,6]. These combined effects enhance scaffold durability and promote the development of uniform pore structures throughout the aerogel network. More importantly, the resulting hierarchical porosity and increased interfacial density establish abundant scattering interfaces and tortuous transfer pathways, which are beneficial for phonon scattering, heat-transfer regulation, and mechanical load redistribution. Therefore, the optimized CA/APP@ATH–MEL aerogel exhibits a clear structure–property relationship between pore architecture, thermal insulation, and mechanical robustness.

#### 3.1.3. Analysis of Micromorphology and Structure–Property Relationship

The microstructural characteristics and pore architecture of the aerogels were systematically investigated by SEM and nitrogen adsorption–desorption measurements, as shown in Figure 3. SEM observations (Figure 3a–c) reveal that pristine CA exhibit relatively ordered lamellar structures; however, numerous irregular and poorly supported pores are present between adjacent layers because of insufficient crosslinking. The weak hydrogen bonding and van der Waals interactions among cellulose chains are unable to withstand the mechanical stresses induced by ice-crystal growth during freeze-drying, leading to partial structural collapse and pore irregularity. After the introduction of ATH, in situ generated inorganic nodes establish short-range coordination interactions with cellulose chains, thereby significantly enhancing both inter- and intramolecular crosslinking [3,39,40]. This reinforced network improves structural integrity and effectively suppresses ice-crystal-induced damage. Furthermore, the incorporation of APP@ATH–MEL introduces abundant functional groups capable of hydrogen bonding, ionic interactions, and condensation reactions, resulting in a highly interconnected three-dimensional network with enhanced interlamellar bridging. Uniformly dispersed nanoparticles also act as structural fillers, stabilizing pore walls and preserving continuous pore channels. Consequently, the aerogel framework evolves from smooth lamellar structures with large voids (CA) to rough, compact, and highly interconnected architectures (CA/APP@ATH–MEL), characterized by increased surface roughness, enhanced vertical supports, and well-defined pore bridges. This microstructural evolution reflects the transformation from weakly bonded lamellae into a robust multiscale crosslinked network.

Nitrogen adsorption–desorption analysis further confirms these structural differences. The adsorption–desorption isotherms exhibit typical type IV behavior with distinct hysteresis loops, indicating the presence of mesoporous structures, while the pore size distribution further confirms a dominant mesopore regime and the formation of hierarchical pore architectures. For the unoptimized system, the BET surface area decreases from 3.87 m^2^·g^−1^ for pristine CA to 1.44 m^2^·g^−1^ after filler incorporation, accompanied by an increase in average pore size and a reduction in pore volume. This behavior can be attributed to partial pore blocking and local structural densification caused by the incorporation of inorganic and hybrid particles. After optimization of the sol–gel processing conditions, including precursor dispersion, controlled aging, and scaffold stabilization, the composite aerogel exhibits a significant increase in surface area to 7.95 m^2^·g^−1^, nearly doubling that of pristine CA. This result indicates that the dual-filler strategy involves a competitive process between initial pore blocking and subsequent network stabilization. Although filler incorporation initially reduces the accessible pore volume, the formation of a robust multiscale crosslinked framework effectively prevents structural collapse during freeze-drying [41,42,43], thereby generating stable mesoporous structures and increased interfacial area. In the unoptimized state, the accessible surface area may decrease because of partial pore occupation and local densification. After optimization of precursor dispersion and sol-aging conditions, however, the stabilized multiscale framework suppresses collapse during freeze-drying and yields a higher preserved surface area. Therefore, the hierarchical pore structure plays a critical role in achieving decoupled heat transfer behavior, consistent with the excellent thermal insulation performance observed in this work. Overall, the proposed dual-filler strategy enables precise regulation of pore architecture and network topology, establishing a clear structure–property relationship among multiscale porosity, thermal transport behavior, and mechanical stability.

### 3.2. Mechanical Reinforcement Through Crosslinking-Induced Network Regulation

**Figure 4:**
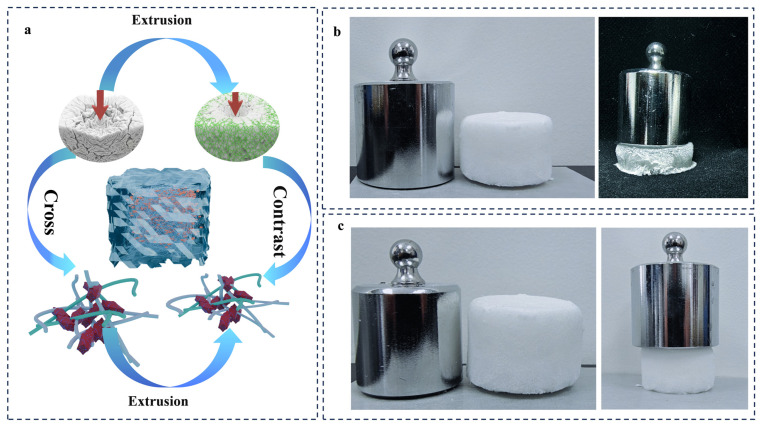
Mechanical-performance comparison of the aerogels: (**a**) schematic illustration of the structural evolution related to compression resistance; (**b**) optical image of pristine CA under compressive loading; and (**c**) optical image of CA/APP@ATH–MEL under compressive loading, showing improved dimensional stability of the composite aerogel.

Although cellulose-based aerogels generally exhibit high porosity and large specific surface area, their practical application is often restricted by structural brittleness and limited resistance to external compression. In the present work, this limitation is alleviated through the combined effect of the pre-sol–gelation/sol-aging strategy and the introduction of nanoscale flame-retardant components, both of which contribute to the formation of a more stable porous framework. As shown in Figure 4a, the incorporation of ATH and APP@ATH–MEL modifies the pore morphology and overall framework organization of the aerogel. Pristine CA exhibits loosely connected lamellar structures with relatively large interlayer voids. Under external loading, such a framework is more prone to local stress concentration and structural collapse. After the introduction of ATH and APP@ATH–MEL, the aerogel develops a more integrated three-dimensional architecture. Interlamellar bridging features, vertical supports, and locally curved structural elements can be observed more clearly, indicating improved structural continuity within the porous framework.

This structural evolution is closely related to the enhanced resistance to compression-induced deformation observed in the composite aerogel. The more continuous interlamellar connections facilitate stress transfer between adjacent lamellae, while the supporting structural features help reduce local stress concentration. At the same time, the more compact and better-preserved pore architecture makes the framework less susceptible to collapse during compression. As illustrated in Figure 4b,c, the composite sample maintains its shape more effectively than pristine CA under loading, which qualitatively reflects improved dimensional stability. At the molecular and mesoscale levels, this behavior is consistent with stronger particle–matrix interactions and improved framework stabilization. ATH-related species can provide rigid inorganic support within the cellulose scaffold, whereas APP@ATH–MEL contributes additional interaction sites and structural reinforcement effects. Their combined presence appears to promote a more stable load-bearing framework and to suppress the collapse of lamellar regions during deformation. In this sense, the mechanical improvement of the composite aerogel is not simply associated with filler addition, but with the regulation of pore morphology and framework connectivity throughout the structure. It should also be noted that the photographic demonstrations in Figure 4b,c involve samples of different dimensions. The pristine CA sample has a diameter of 3 cm, a height of 2 cm, and a mass of approximately 3 g, whereas the CA/APP@ATH–MEL sample has a diameter of 5 cm, a height of 4 cm, and a mass of approximately 6 g. Therefore, these images are intended primarily as qualitative evidence of improved resistance to compression-induced collapse rather than as a strict quantitative comparison of compressive strength. Even so, the visibly improved dimensional stability of the composite sample remains consistent with the formation of a more stable hierarchical porous framework. the mechanical behavior of the composite aerogel can be understood in terms of pore-structure regulation and framework stabilization. A more continuous and better-preserved architecture helps the material resist local collapse under load. This result further indicates that, in cellulose-based composite aerogels, pore morphology is a key factor governing compression resistance and structural integrity.

### 3.3. Thermal Stability and Decomposition

**Figure 5:**
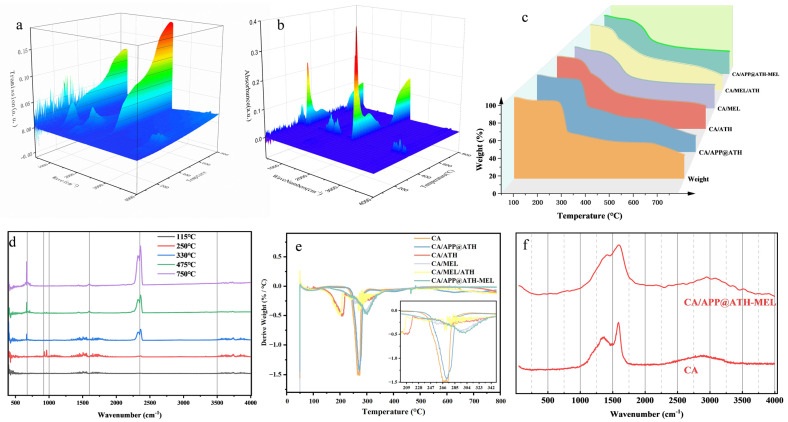
Thermal degradation behavior of pristine CA, CA/ATH, and CA/APP@ATH–MEL aerogels under nitrogen atmosphere: (**a**) TGA curves; (**b**) DTG curves; and (**c**–**e**) TG–FTIR spectra collected at representative temperatures, showing the evolution of volatile species during thermal decomposition; (**f**) Raman spectra of the residual char obtained after combustion.

The thermal stability and fire-related degradation behavior of the aerogels were investigated by thermogravimetric analysis (TGA), derivative thermogravimetry (DTG), and coupled TGA–FTIR under a nitrogen atmosphere (Figure 5). As shown in Figure 5a, pristine CA exhibits an initial mass-loss event at approximately 228 °C, which is attributed to the removal of bound water, followed by a major decomposition stage between 233 and 276 °C associated with cellulose chain scission, depolymerization, and carbonization. The corresponding DTG curve (Figure 5b) shows two characteristic peaks centered at approximately 200 and 290 °C, which are assigned to dehydration and main-chain pyrolysis, respectively. After this process, the residual char yield is about 29%. After the incorporation of ATH, the composite aerogel retains a similar two-stage degradation profile, although the decomposition behavior becomes slightly delayed, indicating that ATH alone provides only limited thermal stabilization. By contrast, the CA/APP@ATH–MEL aerogel shows a shift in the DTG peaks toward higher temperatures together with a decrease in the maximum mass-loss rate from 0.47 to 0.40%·min^−1^. At the same time, the final char yield increases from 28% for pristine CA to 36% for the composite aerogel. These results indicate that the combined flame-retardant system moderates thermal degradation and promotes residue formation. It is noteworthy that the initial decomposition temperature (Td_5_%) decreases from 237 °C for pristine CA to 167 °C for the composite aerogel. This change should not be interpreted simply as reduced thermal stability. In the present system, the earlier mass loss is more reasonably associated with the endothermic dehydration of ATH and the activation of phosphorus-containing species. These early-stage processes can absorb heat and promote the formation of protective condensed-phase structures before the main degradation of the cellulose matrix, thereby contributing to improved thermal resistance at later stages. TGA–FTIR analysis (Figure 5c–e) provides further information on the evolution of volatile products during decomposition. At 115 °C, weak absorption bands related to –OH vibrations and initial phosphate-containing species are observed. At 250 °C, the intensity of the CO_2_-related band near 2350 cm^−1^ increases significantly, suggesting the early decomposition of phosphorus-containing groups and the onset of gas-phase dilution effects. Meanwhile, bands in the range of 1330–1360 cm^−1^ indicate the formation of phosphate-related condensed-phase structures. At 330 °C, characteristic absorption associated with ATH decomposition suggests the release of water vapor, which can contribute to gas-phase dilution and may also facilitate condensed-phase reactions between cellulose hydroxyl groups and phosphorus-containing species. As the temperature increases to 475 °C, strong CO and CO_2_ signals appear in the 1250–1750 cm^−1^ region, corresponding to advanced carbonization. However, the composite aerogel exhibits weaker volatile intensity than pristine CA, indicating reduced volatile release during thermal degradation. At 750 °C, the further weakened gas-phase signals are consistent with the formation of a more stable residual layer that suppresses volatile transport. In summary, the improved thermal behavior of the composite aerogel can be attributed to the synergistic action of early-stage endothermic shielding, gas-phase dilution through the release of CO_2_ and H_2_O, and condensed-phase residue formation [2,44,45]. These combined effects help suppress heat and mass transfer during decomposition and are consistent with the improved flame-retardant behavior of the composite system.

### 3.4. Fire Behavior and Flame-Retardant Performance

**Figure 6:**
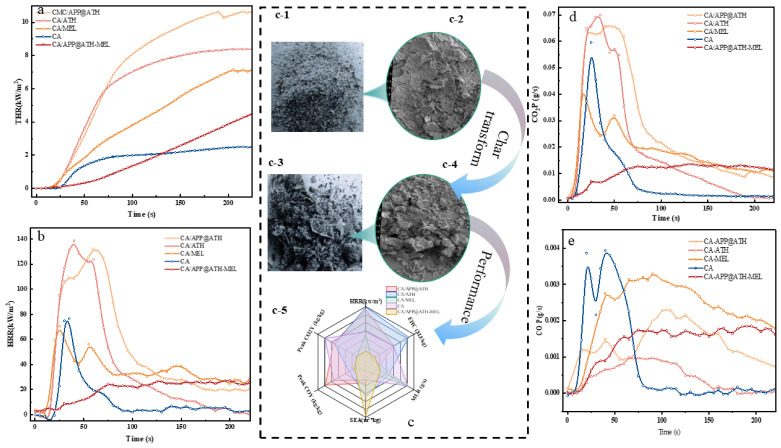
Cone calorimetry (CCT) results of CA, CAATH, and CA/APP@ATH-MEL aerogels under an external heat flux of 35 kW·m^−2^. (**a**) Heat release rate (HRR) curves, (**b**) total heat release (THR), and (**c-1**–**c-5**) smoke and gaseous emission behavior (COY, CO_2_Y, and SEA), (**d**) CO_2_ production rate and (**e**) CO production rate curves of different cellulose aerogel composites during cone calorimeter tests.

The fire behavior and flame-retardant performance of the aerogels were evaluated by cone calorimetry (CCT) under an external heat flux of 35 kW·m^−2^ (Figure 6). As shown in Figure 6a,b, CA exhibits a relatively lower HRR than some single-additive samples. The HRR behavior of the single-additive systems should be interpreted with caution. Although APP, ATH, and APP@ATH can release nonflammable gases during decomposition, HRR is not governed by gas-phase dilution alone. It is also affected by pore-structure integrity, residue continuity, heat feedback, and volatile transport. In the single-additive samples, the additives may disturb the original CA framework or generate discontinuous residues, thereby facilitating heat penetration and combustible-volatile release. APP-derived acidic species may also promote earlier cellulose dehydration/decomposition, while ATH dehydration may introduce local defects if the inorganic residues are not incorporated into a compact protective layer. As a result, the dilution effect of nonflammable gases may be insufficient to reduce HRR. In contrast, CA/ATH/APP@ATH–MEL provides a more balanced gas-phase and condensed-phase protection, leading to reduced HRR and THR. In addition, the reduced CO and CO_2_ release shown in Figure 6d,e indicates lower gaseous-emission intensity during combustion. Meanwhile, the increased specific extinction area (SEA) suggests a more prolonged smoke-evolution process, which is more consistent with regulated decomposition and residue stabilization than with abrupt flame propagation.

The improved fire performance of the composite aerogel can be understood in terms of the synergistic action of gas-phase and condensed-phase processes. During combustion, APP@ATH–MEL can release nonflammable gases, mainly H_2_O and NH_3_, which contribute to gas-phase dilution. The CO_2_ signal in Figure 6d is more complex, as it may arise from both oxidation of combustible fragments and decomposition of flame-retardant components. Therefore, the CO_2_ trend should not be interpreted simply as reduced gaseous emission, but rather as part of the overall combustion and dilution process. At the same time, phosphorus-containing components promote the formation of a more protective condensed-phase residue. ATH decomposition further contributes inorganic species that help stabilize the residual layer. As shown in Figure 6c(c-1–c-5), the composite aerogel forms a more compact and cohesive post-combustion char structure, whereas pristine CA leaves a comparatively fragile and discontinuous residue. This difference in residue morphology is important, because a more stable residual layer can help reduce heat feedback, oxygen ingress, and volatile escape during continued combustion. In addition, the regulated hierarchical framework introduces more tortuous pathways for heat and mass transport, which further supports the suppression of flame propagation. Taken together, the above results show that the CA/APP@ATH–MEL aerogel exhibits improved fire safety, as demonstrated by reduced heat release, lower gaseous-emission intensity, and better residue stability. These observations provide a basis for further elucidating the underlying flame-retardant mechanism, which will be discussed in the following section.

### 3.5. Thermal Insulation and Flame-Retardant Mechanism

Aerogels are well known for their thermal-insulation performance because of their ultralow density, high porosity, and tortuous pore structure. In the CA/APP@ATH–MEL system, these intrinsic advantages are further enhanced by the formation of a hierarchical pore architecture together with the incorporation of inorganic–organic flame-retardant components. The composite aerogel exhibits a low thermal conductivity of 35 mW·m^−1^·K^−1^, which is lower than CA (41 mW·m^−1^·K^−1^), conventional polyethylene foam and glass fiber mats and approaches the performance of advanced cellulose nanofiber aerogels. This behavior is closely related to the stabilized three-dimensional porous framework and the BET surface area of 7.95 m^2^·g^−1^, both of which provide abundant interfaces for phonon scattering and help suppress heat transfer. In addition to passive insulation, the composite aerogel also shows an active thermal-shielding effect during heating. ATH decomposition releases water vapor through an endothermic process, thereby dissipating thermal energy and delaying heat penetration [46,47]. At the same time, APP@ATH–MEL generates phosphorus-containing species that promote dehydration and favor the formation of a more protective condensed-phase residue. The interconnected micro-/nanoporous structure also suppresses gas convection and enhances radiative scattering, leading to a more tortuous heat-transfer pathway.

**Figure 7:**
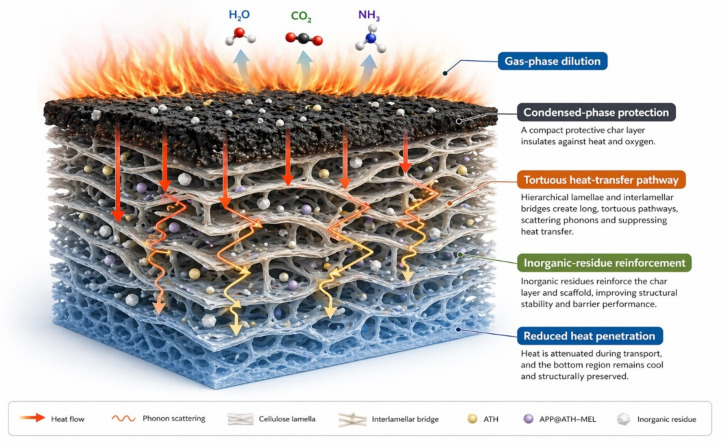
Schematic illustration of the thermal-insulation and multi-phase flame-retardant mechanism of the CA/APP@ATH–MEL composite aerogel. The regulated hierarchical pore structure suppresses heat transfer under normal conditions, while endothermic dehydration, gas-phase dilution, condensed-phase residue formation, and inorganic reinforcement together enhance fire safety under thermal exposure. This figure was generated using AI Image Enhancer (Chatgpt 5.5).

Structural characterization after combustion (Figure 7) shows that the composite aerogel retains a relatively well-preserved porous carbon framework. Raman analysis indicates an increased degree of graphitization, suggesting that MEL contributes to the formation of more thermally stable carbonaceous structures during pyrolysis. The resulting carbon skeleton exhibits thicker pore walls and improved structural integrity, while residual micro-/nanopores continue to act as diffusion barriers that limit oxygen ingress and volatile release. The performance of the composite aerogel can be understood in terms of a synergistic multi-phase flame-retardant mechanism. A denser residual layer provides condensed-phase protection and helps reduce heat transfer and thermal feedback [48,49,50]. Gas-phase protection arises from the release of nonflammable species, including H_2_O, NH_3_, and CO_2_. In addition, phosphorus-containing species and inorganic residues jointly contribute to the stabilization of the char structure. Together, these effects help the aerogel maintain structural integrity and low thermal conductivity under thermal exposure. The results indicate that combining hierarchical pore regulation with multi-phase flame-retardant action is an effective strategy for developing fire-safe thermal-insulation aerogels. The flame-retardant behavior of the composite aerogel arises from the combined contribution of several components. ATH and APP serve as the major flame-retardant constituents, whereas PA and MEL contribute to char promotion and gas-phase dilution effects. Ammonia mainly regulates the alkaline environment required for ATH formation, and the silane-based crosslinker primarily improves structural stability, which may indirectly enhance fire performance by stabilizing the condensed-phase residue [51,52,53].

## 4. Conclusions

A cellulose-based composite aerogel with improved mechanical stability, thermal insulation, and fire safety was fabricated through a multiscale network-regulation strategy. The synergistic incorporation of in situ generated ATH and microencapsulated APP@ATH–MEL not only introduced flame-retardant functionality, but also promoted framework preservation and hierarchical pore regulation during freeze-drying. As a result, the optimized composite aerogel exhibited a low thermal conductivity of 35 mW·m^−1^·K^−1^, improved compressive robustness, moderated thermal degradation, reduced heat release, lower gaseous-emission intensity, and enhanced post-combustion residue stability.

The improved performance is attributed to the combined effects of endothermic shielding, gas-phase dilution, condensed-phase protection, and inorganic-residue reinforcement. Nevertheless, several limitations remain. More direct evidence is still needed to clarify specific interaction mechanisms, the structure–property relationship should be quantified more systematically, and the long-term performance of the aerogel under practical service conditions requires further evaluation. In addition, the applicability of the material may be constrained by factors such as moisture sensitivity, mechanical durability under cyclic loading, and scalability of the fabrication process, which could limit its use in harsh or large-scale industrial environments. These results indicate that hierarchical pore regulation coupled with multi-phase flame-retardant design is an effective strategy for developing lightweight, fire-safe cellulose-based composite aerogels for thermal-insulation applications.

## Data Availability

The original contributions presented in this study are included in the article/Supplementary Materials. Further inquiries can be directed to the corresponding authors.

## Supplementary Materials

The following supporting information can be downloaded at: https://www.mdpi.com/article/10.3390/ma19143106/s1, Table S1: Flame-retardant precursor and suspension formulations; Table S2: summarizes the nominal compositions of the aerogel samples based on precursor feed and processing route; Table S3: BET surface area, average pore size, and pore volume of the initial/unoptimized cellulose-based aerogel system; Table S4: Comparison of representative cellulose-based aerogels in terms of thermal conductivity and flame-retardant performance. References [54,55,56,57,58] are cited in the Supplementary Materials.
