# Supplementary material for: Engineering Hierarchical Cellulose Aerogel Networks Toward Decoupled Heat Transfer and Enhanced Multi-Phase Fire Safety
Source: Materials (Basel). 2026 Jul 20;19(14):3106. doi: 10.3390/ma19143106 (PMC13413962; doi:10.3390/ma19143106)
Supplement: Supplementary file 1 [file materials-19-03106-s001.zip › materials-4276876-supplementary.pdf]

Table S1: Flame-retardant precursor and suspension formulations

Code	System / intermediate	Main components	Nominal feed / formulation basis	Main role in final system
**P1**	APP hydrolysis / precursor dispersion	APP, ammonia water, ethanol/water medium	APP **5 g**, ammonia water **2 mL**, water:ethanol = **5:3**, total volume **160 mL**	Provides APP-derived phosphorus source and precursor medium
**P2**	APP@ATH precursor	APP, $AlCl_3 \cdot 6H_2O$, TEOS, CTAB, silane reagent	APP **5 g**, CTAB **0.34 g**, TEOS **1 mL**, $AlCl_3 \cdot 6H_2O$ **4 g**, silane reagent **0.96 g**; water bath at **60 °C for 24 h**	Forms APP@ATH hybrid flame-retardant precursor containing APP-derived and ATH-related components
**P3**	MEL/PA solution	MEL, PA, ultrapure water	MEL **2 g** dissolved in **100 mL** ultrapure water; PA **2 mL** added	Provides nitrogen-containing and phosphorus-containing auxiliary flame-retardant species
**P4**	APP@ATH–MEL suspension	APP@ATH + MEL/PA solution	APP@ATH **1 g** added into MEL/PA solution	Final hybrid flame-retardant suspension used for composite aerogel preparation

Table S2: summarizes the nominal compositions of the aerogel samples based on precursor feed and processing route.

Sample	Cellulose phase	ATH-related component	APP-related component	MEL	PA	Preparation route / direct source	Suggested manuscript description
**CA**	Present	—	—	—	—	Cellulose sol → freezing → freeze-drying	Pristine CMC aerogel

**Sample**	**Cellulose phase**	**ATH-related component**	**APP-related component**	**MEL**	**PA**	**Preparation route / direct source**	**Suggested manuscript description**
**CA/ATH**	Present	Present, generated in situ from $AlCl_3\cdot 6H_2O$ / NH_4OH system	—	—	—	CMC solution + **10 wt% $AlCl_3\cdot 6H_2O$ and ammonia-water mixture** → freezing → drying	CMC aerogel containing ATH-related inorganic species
**CA/MEL**	Present	—	—	Present	Present	**20 mL** cellulose sol (**2 wt%**) + **4 mL** PA/MEL mixture; diluted to **40 mL**; stirred at **60 °C for 30 min** → freeze-drying	CMC aerogel containing MEL/PA system
**CA/APP@ATH**	Present	Present via APP@ATH	Present via APP@ATH	—	—	**20 mL** cellulose sol (**2 wt%**) + APP@ATH-containing suspension → freeze-drying	CMC aerogel containing APP@ATH
**CA/ATH/APP@ATH–MEL**	Present	Present, including in situ ATH and APP@ATH-derived ATH-related species	Present via APP@ATH-derived particles	Present	Present	**20 mL** cellulose sol (**2 wt%**) + **4 mL** APP@ATH–MEL suspension; water bath at **60 °C**, stirring at **1000 rpm for 30 min** → freeze-drying	Final cellulose-based composite aerogel containing ATH and APP@ATH–MEL

Table S3. BET surface area, average pore size, and pore volume of the initial/unoptimized cellulose-based aerogel system.

Sample / state	BET surface area ($m^2 \cdot g^{-1}$)	Average pore size (nm)	Pore volume ($cm^3 \cdot g^{-1}$)	Notes
**CA**	3.8699	3.2975	0.003190	Baseline of the initial/unoptimized system
**CA/APP@ATH–MEL**	1.4421	5.3595	0.001932	Initial filler incorporation led to reduced accessible surface area and pore volume, but increased average pore size

Table S4. Comparison of representative cellulose-based aerogels in terms of thermal conductivity and flame-retardant performance.

System	Main strategy / composition	Thermal conductivity	Representative flame-retardancy metric(s)	Key feature / comment
This work: CA/ATH/APP@ATH–MEL	In situ ATH + APP@ATH–MEL in cellulose scaffold	35 $mW \cdot m^{-1} \cdot K^{-1}$	Reduced HRR and THR; lower CO/CO_2 emission; improved residue stability	Add your exact Figure 6 values in the final manuscript
CNF/DOPO-IA composite aerogel[1]	Phosphorus-containing DOPO-IA modification	28.6–31.2 $mW \cdot m^{-1} \cdot K^{-1}$	pHRR reduce 67%, THR reduce 60%; char residue 19.8 wt% at 700 °C	Strong balance of low thermal conductivity and marked cone-calorimetry

System	Main strategy / composition	Thermal conductivity	Representative flame-retardancy metric(s)	Key feature / comment
				improvement
Fully biobased cellulose/chitosan aerogel (LBL6)[2]	Cellulose filaments/chitosan + phytic acid/chitosan layer-by-layer coating	<38.2 $mW\cdot m^{-1}\cdot K^{-1}$	LOI 63%, pHRR 6.0 $kW\cdot m^{-2}$, THR 0.4 $MJ\cdot m^{-2}$	Very strong fire performance with low thermal conductivity in a fully biobased system
TPMPAT/CNF or PDMS-TPMPAT/CNF aerogel[3]	P/N-containing TPMPAT flame retardant, with optional PDMS hydrophobization	Comparable to commercial polymeric aerogels	UL-94 V-0; self-extinguishing behavior; pure CNF baseline LOI 23.0%	Good combined fire resistance and water resistance; exact k not reported in the accessible abstract snippet
CNF/CA/BA anisotropic composite aerogel[4]	CNF + calcium alginate + boric acid, directional freeze-drying	~30 $mW\cdot m^{-1}\cdot K^{-1}$	LOI 44.2%, UL-94 V-0, low heat release	Intrinsically flame-retardant crosslinking system with low k and good strength

System	Main strategy / composition	Thermal conductivity	Representative flame-retardancy metric(s)	Key feature / comment
MgAl-LDH/cellulose composite aerogel[5]	MgAl-LDH nanosheets as green flame-retardant nanofiller	NR in accessible abstract snippet	pHRR reduced by 41% (CAC) and 50% (CAP) vs neat cellulose aerogel	Good comparative row for flame-retardancy improvement when k is not available in abstract data

[1] Huang, J.; et al. Eco-friendly thermally insulating cellulose aerogels with exceptional flame retardancy, mechanical property and thermal stability. *Case Stud. Therm. Eng.* **2022**.

[2] Varamesh, A.; Zhu, Y.; Hu, G.; Wang, H.; Rezania, H.; Li, Y.; Lu, Q.; Ren, X.; Jiang, F.; Bryant, S. L.; Hu, J. Fully biobased thermal insulating aerogels with superior fire-retardant and mechanical properties. *Chem. Eng. J.* **2024**, *495*, 153587. https://doi.org/10.1016/j.cej.2024.153587.

[3] Guo, W.; Chen, S.; Liang, F.; Fei, B. Ultra-light-weight, anti-flammable and water-proof cellulosic aerogels for thermal insulation applications. *Int. J. Biol. Macromol.* **2023**.

[4] Tu, J.; Mao, T.; Xie, S.; Xiao, H.; Wang, P. Lightweight, ultrahigh-strength and flame-retardant cellulose aerogel crosslinked with a reactive P/N-rich curdlan derivative. *Int. J. Biol. Macromol.* **2024**. https://doi.org/10.1016/j.ijbiomac.2024.135991.

[5] Wu, X.; Liu, M.; Xie, L.; et al. Mechanically robust cellulose nanofiber/sepiolite aerogel composites with superior flame retardant properties. *Cellulose* **2025**, *32*(3), 1685–1698. https://doi.org/10.1007/s10570-025-06374-5.
